# Attention-Deficit/Hyperactivity Disorder Animal Model Presents Retinal Alterations and Methylphenidate Has a Differential Effect in ADHD versus Control Conditions

**DOI:** 10.3390/antiox12040937

**Published:** 2023-04-15

**Authors:** Eliane S. Sanches, Raquel Boia, Ricardo A. Leitão, Maria H. Madeira, Carlos A. Fontes-Ribeiro, António Francisco Ambrósio, Rosa Fernandes, Ana Paula Silva

**Affiliations:** 1 Institute of Pharmacology and Experimental Therapeutics, Faculty of Medicine, University of Coimbra, 3000-548 Coimbra, Portugal; 2 Coimbra Institute for Clinical and Biomedical Research (iCBR), Faculty of Medicine, University of Coimbra, 3000-548 Coimbra, Portugalmariah.madeira@gmail.com (M.H.M.);; 3 Center for Innovative Biomedicine and Biotechnology (CIBB), University of Coimbra, 3004-531 Coimbra, Portugal; 4 Clinical Academic Center of Coimbra (CACC), 3004-561 Coimbra, Portugal; 5 Association for Innovation and Biomedical Research on Light and Image (AIBILI), 3000-548 Coimbra, Portugal

**Keywords:** attention-deficit/hyperactivity disorder, methylphenidate, neuroinflammation, retina, visual function

## Abstract

Attention-Deficit/Hyperactivity Disorder (ADHD) is one of the most prevalent neurodevelopmental disorders. Interestingly, children with ADHD seem to experience more ophthalmologic abnormalities, and the impact of methylphenidate (MPH) use on retinal physiology remains unclear. Thus, we aimed to unravel the retina’s structural, functional, and cellular alterations and the impact of MPH in ADHD versus the control conditions. For that, spontaneously hypertensive rats (SHR) and Wistar Kyoto rats (WKY) were used as animal models of ADHD and the controls, respectively. Animals were divided into four experimental groups as follows: WKY vehicle (Veh; tap water), WKY MPH (1.5 mg/kg/day), SHR Veh, SHR MPH. Individual administration was performed by gavage between P28-P55. Retinal physiology and structure were evaluated at P56 followed by tissue collection and analysis. The ADHD animal model presents the retinal structural, functional, and neuronal deficits, as well as the microglial reactivity, astrogliosis, blood-retinal barrier (BRB) hyperpermeability and a pro-inflammatory status. In this model, MPH had a beneficial effect on reducing microgliosis, BRB dysfunction, and inflammatory response, but did not correct the neuronal and functional alterations in the retina. Curiously, in the control animals, MPH showed an opposite effect since it impaired the retinal function, neuronal cells, and BRB integrity, and also promoted both microglia reactivity and upregulation of pro-inflammatory mediators. This study unveils the retinal alterations in ADHD and the opposite effects induced by MPH in the retina of ADHD and the control animal models.

## 1. Introduction

Attention-deficit/hyperactivity disorder (ADHD) is a neurodevelopmental condition with a mean worldwide prevalence of 2.2% that often manifests in childhood but can persist in adulthood. This impairing condition creates a substantial burden for the individual, their family, and the community [1]. ADHD is characterized by a persistent pattern of inattention and/or hyperactivity-impulsivity that interferes with the normal functioning and development [2]. Although the exact etiology of ADHD remains unclear, neurodevelopmental, genetic, and environmental factors have been implicated. Additionally, over the past few years, both clinical and preclinical studies have demonstrated structural and functional brain alterations in ADHD [3,4]. At the cellular level, the dopaminergic system has been the main target of study in this field due to the treatment efficacy of stimulants.

Methylphenidate (MPH) is the first-line pharmacological treatment for ADHD and has been used worldwide based on its ability to relieve core target symptoms of this disorder [5]. The primary pharmacodynamic effects of MPH are described as blocking the reuptake of dopamine (DA) and norepinephrine (NE) into the presynaptic terminals through the inhibition of the DA and NE transporters (DAT and NET, respectively) [5]. However, other effects of MPH on the Central Nervous System (CNS) were previously identified by us, particularly on brain endothelial and glial cells [4,6,7], raising the importance of an in-depth investigation of the impact of this widely used psychostimulant. In fact, we have previously demonstrated that in ADHD conditions (spontaneously hypertensive rats, SHR), MPH (1.5 mg/kg/day) had a beneficial effect by attenuating neuroinflammation. In contrast, in the control rats (WKY) MPH induced BBB dysfunction, a robust neuroinflammatory and oxidative response [4].

The retina is a thin layer of tissue lining the back of the eye and is considered part of the CNS. In recent years, the concept of the retina as a possible mirror of brain changes has been claimed in several studies showing retinal alterations in both neurodegenerative diseases [8,9] and psychiatric conditions [10,11,12]. A link between ADHD and vision problems has already been established in some studies. It has been reported that children with vision problems including color vision deficiency, monocular vision loss, and strabismus have a higher prevalence of ADHD [13]. However, these changes cannot be corrected with the use of glasses or contact lens [13] and very little is known about retinal changes associated with ADHD. Nazari and collaborators [14] demonstrated an early deficit in visual sensory integration using event-related potentials measured in the visual cortex of ADHD children, showing the potential relationship between vision and ADHD. On the other hand, it was also reported that some cases of ADHD misdiagnosis might be explained by inattention due to vision problems [13,15], which, if detected and corrected, would avoid the need for pharmacological intervention directed at ADHD. Importantly, in ADHD adult patients, it was demonstrated that the treatment with MPH normalizes the increased retinal background noise observed in these patients [16]. Still, nothing is known about the impact of MPH on the retina in early life.

Regarding preclinical studies, in two different animal models of ADHD, the superior colliculus exhibited structural alterations and increased collicular visual responsiveness that were associated with increased distractible behavior in these animals [17,18]. Indeed, the superior colliculus is a midbrain sensory structure that plays a crucial role in eye and head movements and is associated with distractibility, which is a central component of the clinical features of ADHD. We have also previously shown that an ADHD animal model presents a basal neuroinflammatory status in the brain and the pharmacological treatment with MPH has a beneficial effect by counteracting neuroinflammation [4]. Interestingly, we also found that MPH had a detrimental effect in the control rats since it triggered blood-brain barrier (BBB) dysfunction and induced a robust neuroinflammatory and oxidative response [4]. Curiously, MPH administered to the control rats also interferes with neuronal responses to light stimuli in the dorsal lateral geniculate nucleus, the primary thalamic relay for visual information from the retina to the visual cortex [19]. 

Despite its prevalence, a lack of clear understanding of ADHD pathogenesis with implications on its proper treatment still exists. Since some features commonly associated with ADHD can also be mimicked by visual impairment [15], the characterization of these visual deficits will avoid misinterpretations of ADHD symptoms and, consequently, a more accurate diagnosis. Moreover, MPH has been widely used as the first-line pharmacological treatment for ADHD, but information regarding its impact on vision has been overlooked. Thus, the aims of our study were to characterize the structural, functional, cellular, and molecular changes in the retina of the ADHD animal model, SHR rats, and simultaneously to unravel the impact of MPH on the retina in both healthy and ADHD conditions. SHR is the most well-characterized and frequently used animal model of ADHD [20,21,22]. Prepubertal (4–5 weeks old) and pubertal/young adult SHR (up to 10 weeks of age) exhibit behavioral characteristics of ADHD, including sustained attention deficit, motor impulsiveness, and hyperactivity [20,22,23]. These behavioral ADHD features are related to dysfunction in the fronto-striatal system, since these animals present an impaired dopamine release in the pre-frontal cortex, nucleus accumbens, and caudate-putamen [23]. To date, this is the first study that characterized in detail the retinal alterations during this critical period related to ADHD, as well as the impact of MPH on such changes.

## 2. Materials and Methods

### 2.1. Animals and Treatments

Animals were purchased from Charles River Laboratories (Barcelona, Spain) and housed under controlled environmental conditions (12 h light: dark cycle, 24 ± 1 °C) with food and water ad libitum. The spontaneously hypertensive rat (SHR) is the most well-characterized and commonly used animal model of ADHD [4,23], since it is the model that exhibits neurobiological and behavioral features of this neurodevelopmental condition and so that best fits the criteria for ADHD diagnosis [23]. Moreover, it is a genetic model bred from progenitor Wistar Kyoto rats (WKY) [17], which were used as the control strain. Noteworthy, hypertension in SHR rats develops only in adult animals, and so until late adolescence/young adulthood (up to 10 weeks of age), they only show ADHD features without hypertension symptoms. The animals were randomly distributed into four experimental groups, with 15 animals each, as follows: WKY vehicle, WKY MPH, SHR vehicle, and SHR MPH. Rats received one daily oral administration of MPH (1.5 mg/kg/day by gavage) or vehicle (Veh; tap water) from P28-P57 (equivalent to late childhood through late adolescence in humans). The dose of MPH was selected taking into consideration our previous study, which demonstrated that 1.5 mg/kg of MPH had beneficial effects on the behavior impairment and inflammatory status of SHR animals when compared to a higher dose (5 mg/kg) that had a detrimental effect in both SHR and WKY rats [4]. The animal study protocol was approved by the Institutional Animal Care and Use Committee (FMUC, University of Coimbra, Portugal) and the Portuguese National Authority for Animal Health ‘‘DGAV” (Ref: 0421/000/000/2020). All experiments were performed by certified researchers (Federation for Laboratory Animal Science Associations) in accordance with European Community Council Directives (2010/63/EU) and Portuguese law for the care and use of experimental animals (DL nº 113/2013). The experiments were also in agreement with the guidelines of the Association for Research in Vision and Ophthalmology (ARVO) Statement for Use of Animals in Ophthalmic and Vision Research.

### 2.2. Electroretinogram (ERG) Recordings

ERG recordings were performed after overnight dark adaptation to evaluate retinal physiology using corneal gold wire electrodes as previously described by our group [24,25]. Under dim red light (λ > 600 nm), animals were anesthetized by an intraperitoneal injection with ketamine (90 mg/kg; Nimatek, Dechra, Northwich, UK) and xylazine (10 mg/kg; Sedaxylan, Dechra, Northwich, UK). Then, topical anesthesia with oxybuprocaine (4 mg/mL; Anestocil, Edol, Lisbon, Portugal) and pupillary dilation with tropicamide (10 mg/mL; Tropicil Top, Edol, Lisbon, Portugal) were performed. Celluvisc (1% Carmellose sodium; Allergan SA, Madrid, Spain) was applied between the cornea and the gold ring electrode to improve the conductivity of the generated response. Moreover, a Ganzfeld stimulator (Roland Consult GmbH, Brandenburg an der Havel, Germany) with a very dim blue light flash (0.000095 cd·s/m^2^) was used to evaluate the scotopic threshold response (STR), and white light flashes (0.0095 to 9.49 cd·s/m^2^) were applied under scotopic and photopic conditions. The amplitude (μV) of positive STR (pSTR) and negative STR (nSTR) was evaluated to infer on retinal ganglion cells’ (RGCs) response. Moreover, a-wave and b-wave were extracted under scotopic conditions (reflecting rod photoreceptors and bipolar cells response), as well as the amplitude of b-wave under photopic conditions. A photopic flicker test (reflecting cone photoreceptors response) was also performed with bright white flashes (3.00 and 9.49 cd·s/m^2^). An off-line digital filter was applied on STR and b-wave (high-frequency cut-off of 50 Hz) with the RETIport 4.9.8.7 software (Roland Consult GmbH, Brandenburg an der Havel, Germany).

### 2.3. Optical Coherence Tomography (OCT)

After ERG recordings, while the animals were still under anesthesia, cross-sectional images of the retina were acquired using a Micron IV [25] (Phoenix Research Labs, Pleasanton, CA, USA) to evaluate its structure. The pupil was dilated as described above, and celluvisc was instilled in the eye every few minutes to prevent ocular dehydration during anesthesia. Both eyes were imaged to generate ten B-scans above and ten B-scans below the optic nerve. The segmentation of the generated B-scans was performed using the semi-automatic segmentation InSight 2.0.5618 software (Phoenix Research Labs, Pleasanton, CA, USA). For frame analysis, it was used a total of five single OCT scans as follows: the crossing of the optic nerve (ON = 0), the section ON + 3, the section ON + 5, the section ON − 3, and finally, the section ON − 5. Additionally, retinal layers were segmented as follows: RNFL + GCL + IPL = retinal nerve fiber layer + ganglion cell layer + inner plexiform layer; INL = inner nuclear layer (nuclei of bipolar, amacrine, horizontal and Müller cells); ONL = outer nuclear layer (nuclei of photoreceptors); IS/OS = photoreceptor inner and outer segments. The thickness of each segmented layer and the total retinal thickness were determined by the mean value of the five single B-scans.

### 2.4. Immunolabelling

#### 2.4.1. Retinal Cryosections

Retinal cryosections were prepared as previously described [26]. Briefly, cryosections (14 μm) were obtained on a cryostat (Leica CM3050 S, Leica Biosystems, Wetzlar, Germany) and mounted on Superfrost Plus glass slides (Menzel-Gläser; Thermo Scientific, Waltham, MA, USA). The sections were permeabilized with 0.25% Triton X-100 in PBS for 30 min and then blocked with 10% normal goat serum plus 1% bovine serum albumin (BSA) in PBS for 30 min at room temperature (RT) in a humidified environment. The sections were then incubated with the primary antibody (Supplementary Table S1) at 4 °C overnight, followed by incubation with the corresponding secondary antibodies (Supplementary Table S2) for 1 h at RT in the dark. Finally, nuclei were counterstained with Hoechst (1:2000; Sigma, St. Louis, MO, USA) for 1 h at RT. The slices were then mounted with glycergel mounting medium (Dako, Glostrup, Denmark). From each eye, four sections were analyzed across the total retinal length in an inverted fluorescence (Axio Observer.Z1, Zeiss, Oberkochen, Germany) or confocal (Zeiss LSM 710, Oberkochen, Germany) microscope. 

For rhodopsin and VGlut1 immunoreactivity, the results are expressed as the mean fluorescence intensity (arbitrary units) and were determined in the entire retinal section normalized to the respective length by applying the following formula: corrected total fluorescence = (integrated intensity) − (area of picture × mean background) [27]. The arrestin+ and RBPMS+ cells were counted in the entire retinal section and normalized to the respective length. The percentage of responsive microglia (Iba-1^+^ CD68^+^) per total microglia (Iba-1^+^) per image was normalized to the respective retinal section’s length. From each animal, 10 images were obtained and analyzed using Fiji 1.53c software. 

#### 2.4.2. Retina Wholemounts

After transcardiac perfusion of animals, eyes were enucleated and retinas were dissected as flattened wholemounts, as previously reported [28]. Briefly, the retinas were permeabilized with 0.5% Triton X-100, frozen at −80 °C for 15 min, followed by incubation with the primary antibodies (Supplementary Table S1) at 4 °C overnight and secondary antibodies (Supplementary Table S2) for 2 h at RT. Finally, tissue was mounted with the GCL side up with glycergel mounting medium (DAKO). The retina wholemounts were analyzed in a confocal microscope. Vessel integrity was evaluated using isolectin GS-IB4 staining. Vessel coverage by astrocytes was evaluated by staining with antibodies for collagen IV (vessels) and GFAP (astrocytes), as we previously described [29]. The results were calculated using the Fiji software and expressed as the mean of co-localized voxels of 20 photographs from retinal vessels obtained from three different animals for each experimental group [30]. 

### 2.5. Western Blot Analysis

Retinal protein extracts were prepared as previously described [31]. Briefly, samples (20 μg of protein) were separated in 10%, 12%, or 15% sodium dodecyl sulphate-poly(acrylamide) gel electrophoresis (SDS-PAGE), and the proteins were transferred to polyvinylidene difluoride membrane (Millipore, Madrid, Spain). The membranes were blocked in 5% skim milk in Tris-buffered saline (TBS: 137 mM NaCl, 20 mM Tris-HCl, pH 7.6) containing 0.1% Tween-20 (TBS-T) for 1 h at RT. Afterwards, membranes were incubated with the primary antibodies (Supplementary Table S1), followed by incubation with respective alkaline phosphatase-conjugated secondary antibody for 1 h at RT. Protein detection was conducted using ECF™ (GE Healthcare Amersham™, Chalfont Saint Giles, UK) in accordance with the manufacturer’s instructions on Typhoon FLA 9000 (GE Healthcare Bioscience AB, Uppsala, Sweden). Immunoblots were reprobed with glyceraldehyde 3-phosphate dehydrogenase antibody (GAPDH, 1:5000; Life Technologies, Cambridge, UK) to ensure equal sample loading. Digital quantification of band intensity was performed using Image Studio 5.2 software (LI-COR Biosciences, Lincoln, NE, USA).

### 2.6. Statistical Analysis

The normality of the data was assessed with Shapiro-Wilk and Kolmogorov-Smirnov normality tests. Accordingly, data were analyzed using two-way ANOVA followed by Tukey’s test except for the quantification of the GFAP immunostaining in retinal wholemounts that were analyzed by Kruskal-Wallis test followed by Dunn’s post-test, as indicated in figure legends. The results are presented as mean ± standard error of the mean (SEM). Differences were considered significant if *p* < 0.05 and the *n* “n” represents the total number of animals used in each experimental condition. Statistical analysis was performed with the Prism 8.0.1 Software for Windows (GraphPad Software, Inc., San Diego, CA, USA).

## 3. Results

### 3.1. Impact of MPH Treatment on Retinal Structure and Function 

**Figure 1:**
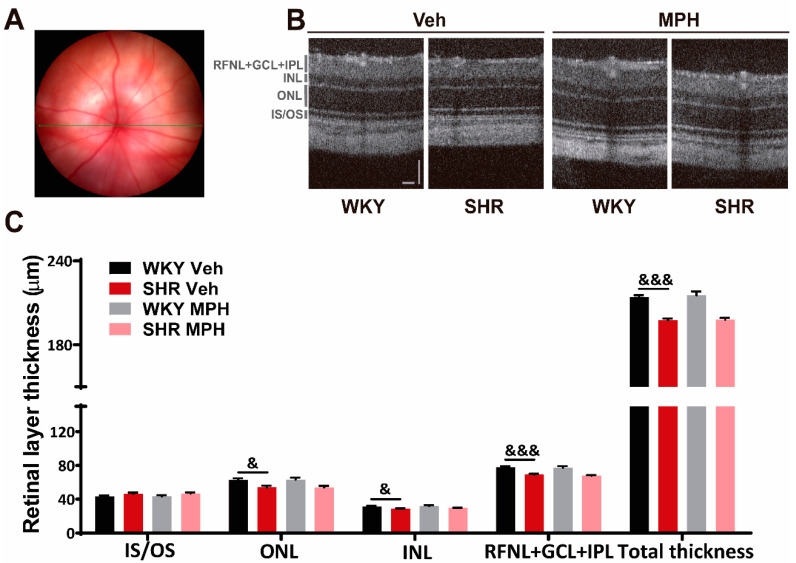
ADHD animal model presents thinning of retinal layers, with no beneficial effect of MPH treatment. (**A**) Representative image of the eye fundus showing the line scan (green line). (**B**) Representative images of in vivo optical coherence tomography (OCT) showing the segmentation of the different retinal layers and the limits considered for the thickness evaluation. Scale bars: 50 μm. (**C**) retinal thickness quantification showing a significant decrease in ADHD conditions in all retinal layers, except in IS/OS. MPH treatment had no effects either on ADHD or on control conditions. Results are presented as mean ± SEM, *n* = 11 animals for each experimental condition. ^&^ *p* < 0.05, ^&&&^ *p* < 0.001, WKY Veh vs. SHR Veh using two-way ANOVA followed by Tukey’s test. RNFL + GCL + IPL = retinal nerve fiber layer + ganglion cell layer + inner plexiform layer; INL = inner nuclear layer; ONL = outer nuclear layer; IS/OS = photoreceptor inner segments and outer segments. WKY = Wistar Kyoto rats; SHR = Spontaneously hypertensive rats; Veh = vehicle; MPH = Methylphenidate.

**Figure 2:**
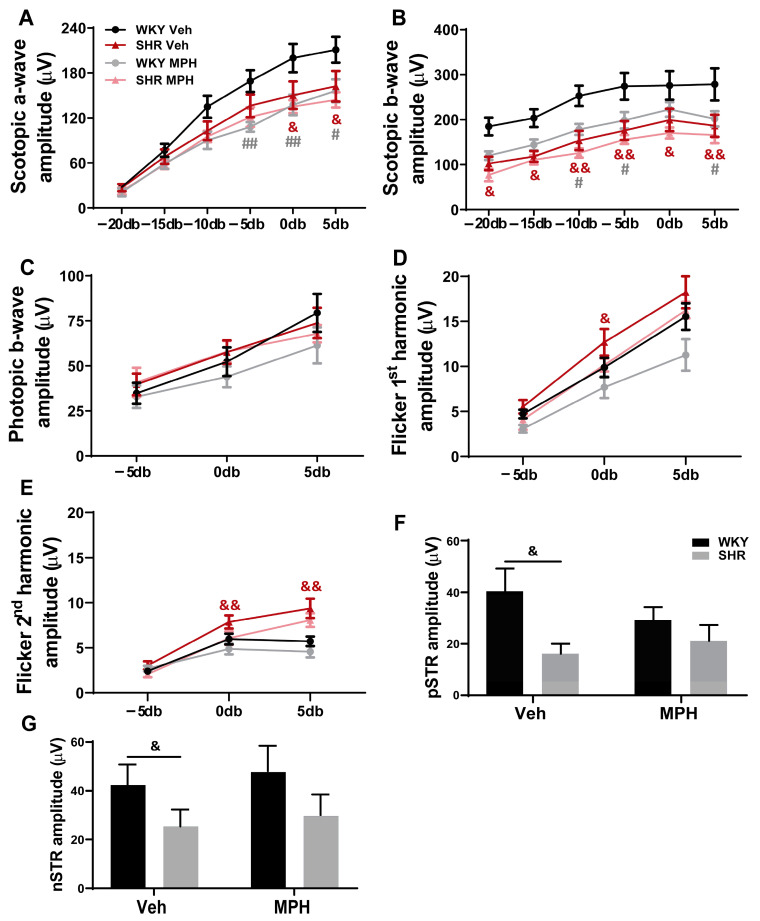
ADHD model presents retinal functional impairment, and MPH treatment per se negatively impacts control rats. Amplitudes of scotopic (**A**) a-wave and (**B**) b-wave, (**C**) photopic b-wave, (**D**,**E**) flicker, (**F**) positive, and (**G**) negative scotopic threshold response (STR). In the ADHD model, MPH treatment did not interfere with the decrease in the photoreceptor, bipolar cells, and retinal ganglion cells response, or the increase in the cone photoreceptors response. On the contrary, MPH per se negatively impacts photoreceptors and bipolar cell responses in control rats. Results are presented as mean ± SEM, *n* = 10–16 animals for each experimental condition. ^&^ *p* < 0.05, ^&&^ *p* < 0.01, WKY Veh vs. SHR Veh; ^#^ *p* < 0.05, ^##^ *p* < 0.01, WKY Veh vs. WKY MPH, using two-way ANOVA followed by Tukey’s test.

To assess possible structural and functional changes in the retina of the ADHD model, we used OCT (Figure 1) and ERG (Figure 2 and Figure S1), non-invasive techniques, respectively. The mean thickness of the total retina and retinal layers was quantified after image segmentation of the inner and outer boundaries (Figure 1C). Overall, the ADHD rat model presented a decrease in the total retinal thickness (197.7 ± 1.1 µm) compared to the WKY rats (213.9 ± 1.6 µm). Regarding the different layers considered in the analysis, the ADHD model presented a decrease in the thickness of several retinal layers when compared with the controls as follows (WKY Veh vs. SHR Veh): 77.6 ± 1.4 vs. 69.3 ± 0.8 µm for RNFL + GCL + IPL, which correspond to nonmyelinated axons of the RGCs that form the optic nerve, the nuclei of ganglion cells and astrocytes, and the synaptic connections between bipolar, amacrine and ganglion cells; 31.4 ± 0.7 vs. 28.5 ± 0.4 µm for INL, which contains the nuclei of bipolar, amacrine, horizontal, and Müller cells that mediate the signal between the photoreceptors and RGCs; and 62.6 ± 2.1 vs. 53.9 ± 2.0 µm for ONL, which is the layer where the nuclei of photoreceptors are located. However, no differences were observed in the IS/OS, which contain visual pigments of the photoreceptors. These results show that the ADHD model presents an impairment in the retinal thickness, namely in the nuclear and plexiform layers. Moreover, the treatment with MPH did not affect the thickness of the retinal layers either of the control or ADHD model. 

Since thickness alterations reflect neural changes and these can have an impact on retinal function, we next used ERG to assess the retinal function of the SHR rats, compared to the controls, both subjected, or not, to the MPH treatment. Considering the maximum light intensity (9.49 cd·s/m^2^, Figure 2A,B), the ADHD model presented a decrease in the amplitude of the scotopic a- and b-wave (162.2 ± 20.4 µV and 186.3 ± 24.4 µV, respectively; Figure 2A,B) compared to the controls (210.9 ± 17.1 µV and 278.4 ± 35.7 µV, respectively), which reflects an impairment in cones and bipolar cell circuits in the dark. However, no significant changes were found in the bipolar cells’ response under photopic conditions (Figure 2C). The MPH treatment per se induced a significant decrease in the scotopic responses in the control rats (156.3 ± 15.2 µV and 201.9 ± 17.0 µV for a- and b-wave, respectively; Figure 2A,B), with no further significant effects on the photopic b-wave amplitude.

The flicker 1st (Figure 2D) and 2nd (Figure 2E) harmonic responses, which are thought to be the most representative of the original waveform [24], were also analyzed to assess the function of cones. The ADHD model presented a significant increase in the 1st (12.7 ± 1.5 µV) and 2nd (9.9 ± 1.1 µV) harmonic amplitude in comparison with the controls (9.4 ± 1.1 and 5.7 ± 0.5 µV, respectively). Finally, we also registered a significant decrease in the RGCs’ function in the ADHD model, as observed in the pSTR (16.3 ± 3.9 µV; Figure 2F) and nSTR (25.4 ± 6.9 µV; Figure 2G) amplitudes compared to the controls (40.6 ± 8.9 µV and 42.2 ± 8.6 µV, respectively). Moreover, no significant effects of the MPH treatment were identified on both cones and RGCs function. In sum, we concluded that ADHD model presented changes in the function of photoreceptors, bipolar cells, and RGCs, and MPH had no effect on these alterations. Concerning control animals, MPH led to an impairment in the physiology of the photoreceptors and bipolar cells.

### 3.2. Neuronal Alterations in the Retina of ADHD Animal Model and the Impact MPH on Control versus ADHD

**Figure 3:**
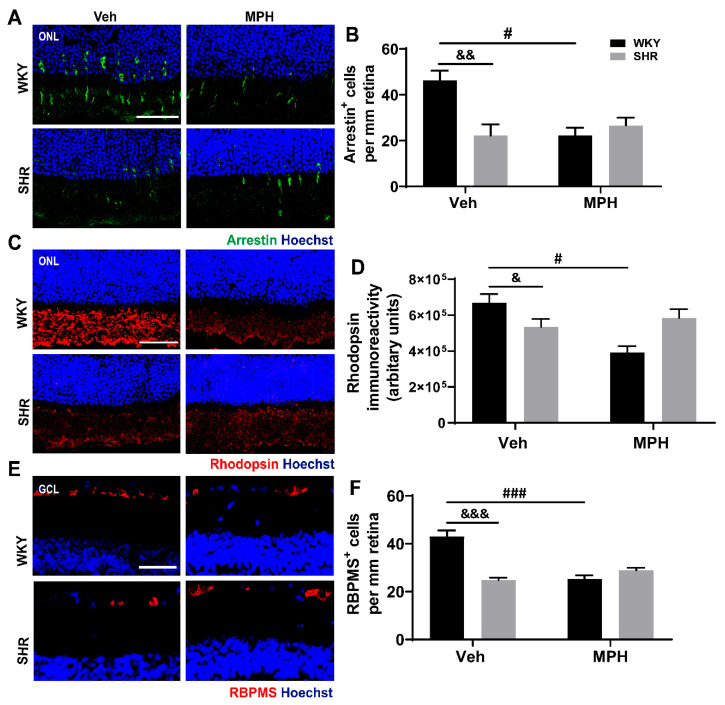
Alterations in retinal neurons in ADHD model and MPH deleterious effects in control animals. (**A**,**C**,**E**) Representative confocal images of retinal cryosections and quantification of (**B**) the number of cones (arrestin, green), (**D**) the immunoreactivity for rhodopsin (rods, red), and (**F**) the number of retinal ganglion cells (RBPMS, RNA binding protein, mRNA processing factor; red). Scale bar: 50 μm. ADHD model presented a decrease in the number of cone photoreceptor and retinal ganglion cells as well as in the immunoreactivity of the rod photoreceptor marker. MPH treatment did not benefit such alterations in the ADHD model, but in control rats, MPH had a negative effect on retinal neurons. Results are presented as mean ± SEM, *n* = 3–5 animals for each experimental condition. ^&^ *p* < 0.05, ^&&^ *p* < 0,01, ^&&&^ *p* < 0.001, WKY Veh vs. SHR Veh; ^#^ *p* < 0.05, ^###^ *p* < 0.001, WKY Veh vs. WKY MPH, using two-way ANOVA followed by Tukey’s test.

Taking into consideration our results regarding retinal structure and function, the number of different retinal neurons was assessed by immunolabelling the retinal cryosections for the cone (Figure 3A,B) and rod (Figure 3C,D) photoreceptors, as well as for the RGCs (Figure 3E,F). The ADHD model (SHR Veh) presented a decrease in the number of cones and RGCs (arrestin+ and RBPMS+ cells; 22.2 ± 4.9 and 24.9 ± 0.9 cells/mm retina, respectively) when compared to the control rats (WKY Veh; 46.2 ± 4.3 and 43.0 ± 2.6 cells/mm retina, respectively) (Figure 3B,F). Moreover, the immunoreactivity for rhodopsin (rods marker) decreased in the ADHD model (6.7 × 10^5^ ± 4.7 × 10^4^ and 5.3 × 10^5^ ± 4.3 × 10^4^ arbitrary units for WKY Veh and SHR Veh, respectively; Figure 3D). Concerning the effect of MPH, it decreased the number (cones and RGCs) and the immunoreactivity (rods) of the different neuronal cells in the control conditions with no effects on the SHR rats (WKY Veh vs. WKY MPH as follows: cones, 46.22 ± 4.31 and 22.20 ± 3.43 cells/mm retina; rods, 6.7 × 10^5^ ± 4.7 × 10^4^ and 3.9 × 10^5^ ± 3.5 × 10^4^ arbitrary units; RGCs, 43.0 ± 2.6 and 25.3 ± 1.5 cells/mm retina. These results indicate that the ADHD model presents significant neuronal changes in the retina, particularly in the photoreceptors and RGCs, and the use of MPH in the WKY animals also had a negative effect on these retinal neuronal cells.

**Figure 4:**
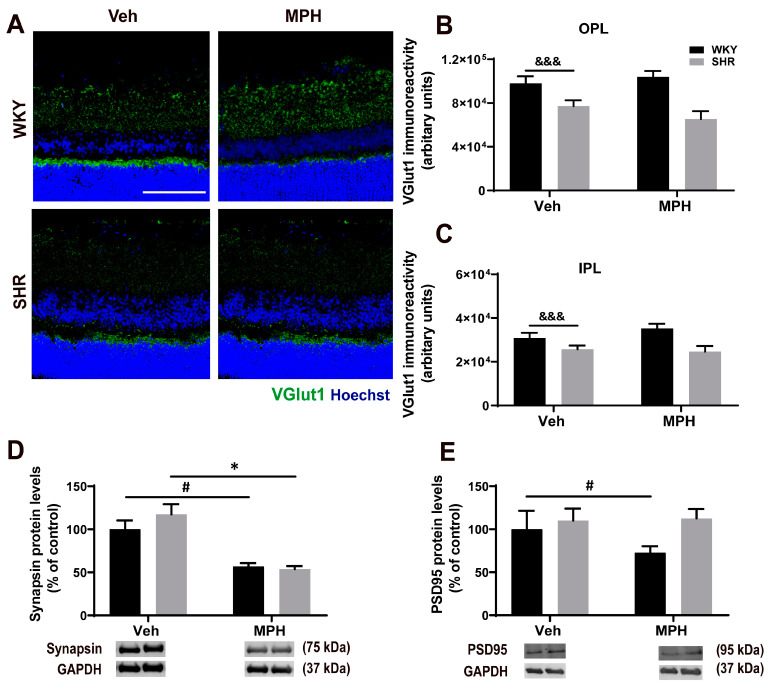
Impact of MPH on synaptic proteins in the retina of the ADHD animal model versus control animals. (**A**) Representative confocal images of retinal cryosections and (**B**,**C**) quantification of VGlut1 (green) positive staining in (**B**) the OPL and (**C**) IPL. Scale bar: 100 µm. ADHD model presented a decrease in VGlut1 staining in both OPL and IPL. MPH treatment had no effects either on ADHD or control conditions. The protein levels of (**D**) the pre-synaptic protein synapsin and (**E**) the post-synaptic density protein 95 (PSD95) were assessed by western blot using retina total extracts. MPH treatment per se significantly decreased the levels of synapsin and PSD95 in control animals and reduced synapsin in the ADHD model. Representative western blot images of synapsin (75 kDa), PSD95 (95 kDa) and GAPDH (37 kDa) are shown. Results are presented as mean ± SEM, *n* = 3–4 animals for each experimental condition. ^&&&^ *p* < 0.001, WKY Veh vs. SHR Veh; ^#^ *p* < 0.05, WKY Veh vs. WKY MPH; * *p* < 0.05, SHR Veh vs. SHR MPH using two-way ANOVA followed by Tukey’s test.

To better characterize the abovementioned functional and neuronal alterations, we further analyzed different synaptic proteins in the outer plexiform (OPL) and inner plexiform layer (IPL). For that, the retinal cryosections were immunolabelled for the vesicular glutamate transporter 1 (VGlut1; Figure 4A–C). The ADHD model (SHR Veh) presented a decrease in VGlut1 immunostaining in the OPL (9.8 × 10^4^ ± 6.43 × 10^3^ vs. 7.7 × 10^4^ ± 5.3 × 10^3^ arbitrary units) and IPL (3.0 × 10^4^ ± 2.3 × 10^3^ vs. 2.5 × 10^4^ ± 2.5 × 10^4^ arbitrary units) as compared to WKY Veh (Figure 4B,C). The treatment with MPH had no effect on both animal strains (SHR and WKY). Furthermore, by western blot, we assessed the levels of the pre-synaptic protein synapsin (Figure 4D) and the post-synaptic density protein 95 (PSD95; Figure 4E), and no differences were detected compared the controls and the ADHD animal model. However, MPH was able to decrease synapsin protein levels in both WKY and SHR (58.8 ± 4.0% and 53.8 ± 3.5% of the control, respectively). Regarding PD95 protein levels, the MPH treatment triggered a decrease, but only in the control rats (72.5 ± 7.7% of the control). The ADHD model presented a decrease in the number of the photoreceptors and RGCs. Moreover, the MPH treatment had deleterious effects in the control rats regarding the number of retinal neurons and also synaptic proteins that could be underlying the deficits observed in retinal function.

### 3.3. Microglia Response and Neuroimmune Profile in ADHD and the Effect of MPH Treatment

**Figure 5:**
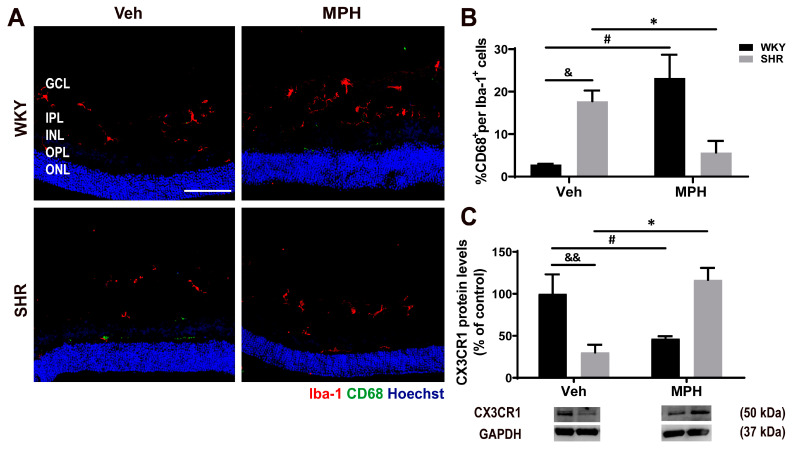
Microglia reactivity in the retina of the ADHD animal model and differential effect of MPH treatment in ADHD and control animals. (**A**) Representative confocal images of retinal cryosections immunostainned for reactive microglial cells (Iba-1, red; CD68, green) and (**B**) quantification of the percentage of reactive microglial cells (Iba-1^+^ CD68^+^) that was normalized to the total microglial cells (Iba-1^+^) per section. Scale bar: 100 µm. (**C**) Quantification of the protein levels of chemokine (C-X3-C) Receptor 1 (CX3CR1). Representative western blot images of CX3CR1 (50 kDa) and GAPDH (37 kDa) are shown. The ADHD model presented an increase in the percentage of reactive microglial cells and a decrease in CX3CR1 levels. MPH treatment had opposite effects in the ADHD model and control rats. Results are presented as mean ± SEM, *n* = 3–4 animals for each experimental condition. ^&^ *p* < 0.05, ^&&^ *p* < 0.01, WKY Veh vs. SHR Veh; ^#^ *p* < 0.05, WKY Veh vs. WKY MPH; * *p* < 0.05, SHR Veh vs. SHR MPH, using two-way ANOVA followed by Tukey’s test.

It has been demonstrated that in retinal degenerative disorders or after an insult, microglial cells that are considered the resident immune cell population, become pathologically activated releasing abnormal amounts of inflammatory mediators that will contribute to tissue damage and disease exacerbation [32]. Thus, we assessed microglia reactivity in the retina of the SHR rats. The percentage of reactive microglial cells (CD68^+^ Iba-1^+^ cells) was significantly increased in the ADHD model (17.7 ± 2.5% and 6.3 ± 3.5% CD68^+^ of Iba-1^+^ cells for SHR Veh and WKY Veh, respectively). The MPH treatment of the SHR animals suppressed the increase in the number of reactive microglia (5.7 ± 2.7% CD68^+^ of Iba-1^+^ cells) (Figure 5A,B). On the contrary, the MPH administration to the control rats (WKY) led to an increase in the percentage of CD68^+^ in Iba-1^+^ cells (23.2 ± 5.5% CD68^+^ of Iba-1^+^ cells).

It has been previously shown that neuronal fractalkine (CX3CL1) acts as a regulator of microglia activation in response to tissue injury or inflammation, since CX3CL1 signals through its unique receptor, CX3CR1, that is expressed in microglia [32]. Thus, changes in the CX3CL1/CX3CR1 pathway, for example by the downregulation of CX3CR1, may promote the reactivity of microglia and stimulate the release of pro-inflammatory factors. Interestingly, we observed that the ADHD animal model presented a downregulation of the CX3CR1 protein levels (30.2 ± 9.1% of the control) in the retina, which was prevented by the MPH treatment (116.7 ± 14.2% of the control). However, in the control rats (WKY Veh), MPH per se had the opposite effect, decreasing the protein levels of CX3CR1 (46.5 ± 3.0% of the control; Figure 5C).

**Figure 6:**
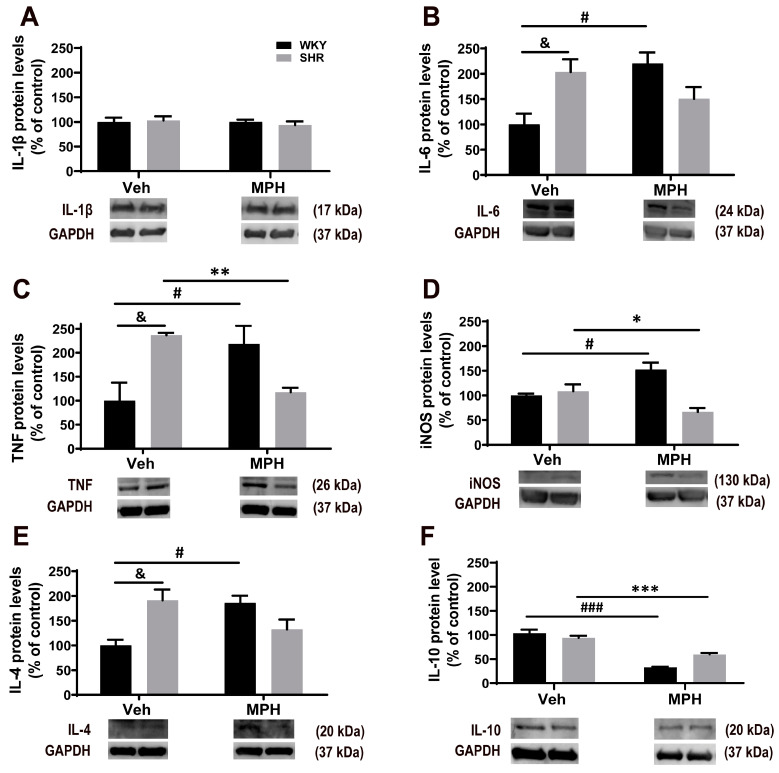
The retina of the ADHD animal model presents a basal inflammatory profile, and MPH treatment induces a distinctive immune response in ADHD versus control animals. Protein levels of (**A**) interleukin (IL)-1 beta (IL-1β), (**B**) IL-6, (**C**) tumor necrosis factor (TNF), (**D**) inducible nitric oxide synthase (iNOS), (**E**) IL-4 and (**F**) IL-10 were assessed by western blot. Representative western blot images of IL-1β (17 kDa), IL-6 (24 kDa), TNF (26 kDa), iNOS (130 kDa), IL-4 (20 kDa), IL-10 (20 kDa), and GAPDH (37 kDa) are also shown. The retina of the ADHD model presents an upregulation of pro-inflammatory mediators, and MPH treatment partially rescues these effects. MPH exposure per se had a negative impact on the retina of control animals by downregulating anti-inflammatory cytokine IL-10 and upregulating pro-inflammatory mediators. Results are presented as mean ± SEM, as % of control, *n* = 3–4 animals for each experimental group. ^&^ *p* < 0.05, WKY Veh vs. SHR Veh; ^#^ *p* < 0.05, ^###^ *p* < 0.001, WKY Veh vs. WKY MPH; * *p* < 0.05, ** *p* < 0.01, *** *p* < 0.001, SHR Veh vs. SHR MPH using two-way ANOVA followed by Tukey’s test.

Having found significant changes in microglia reactivity and knowing that these cells are the main source of inflammatory mediators, we further assessed the levels of different cytokines in the retinal tissue. Regarding the pro-inflammatory cytokine IL-1β, no differences were observed between the experimental groups (Figure 6A). Nevertheless, both IL-6 (Figure 6B) and TNF (Figure 6C) were significantly upregulated in the ADHD animal model (203.6 ± 25.0% and 236.7 ± 4.9% of the control, respectively). In the ADHD animal model, treatment with MPH had no significant effects on the levels of IL-1β and IL-6, but significantly decreased the TNF protein levels (117.40 ± 9.39% of the control). However, the MPH treatment per se increased both IL-6 and TNF protein levels in the control rats (220.4 ± 21.8% and 218.4 ± 37.9% of the control, respectively), with no effect on IL-1β. Moreover, in the ADHD model, the retinal levels of the inducible nitric oxide synthase (iNOS) protein, which is also involved in inflammatory processes, were similar to the control rats (Figure 6D), but the MPH treatment led to a downregulation in the ADHD rats (SHR MPH, 66.7 ± 7.7% of the control). Conversely, in the control rats, MPH upregulated iNOS protein levels in the retina (WKY MPH, 152.5 ± 14.0% of the control).

Concerning anti-inflammatory cytokines, the ADHD animal model also presented a significant increase in the IL-4 levels in the retina (Figure 6E; 191.4 ± 21.6% of the control) compared to the controls (WKY Veh), but the IL-10 levels were not altered (Figure 6F). Treatment with MPH had no significant effect in the SHR rats but increased the levels of IL-4 in the WKY rats (185.92 ± 14.59% of the control; Figure 6E). On the other hand, MPH increased the IL-10 levels both in the WKY and SHR animals (36.3 ± 1.7% and 61.9 ± 6.3% of the control, respectively; Figure 6F). Our data show that the ADHD animal model presents a basal pro-inflammatory status in the retina, and MPH partially rescues this inflammatory profile. However, under normal physiological conditions, MPH has an opposite effect, promoting a pro-inflammatory environment in the retina.

### 3.4. Astrocytic and Retinal Vascular Alterations in ADHD Animal Model and Impact of MPH Treatment

**Figure 7:**
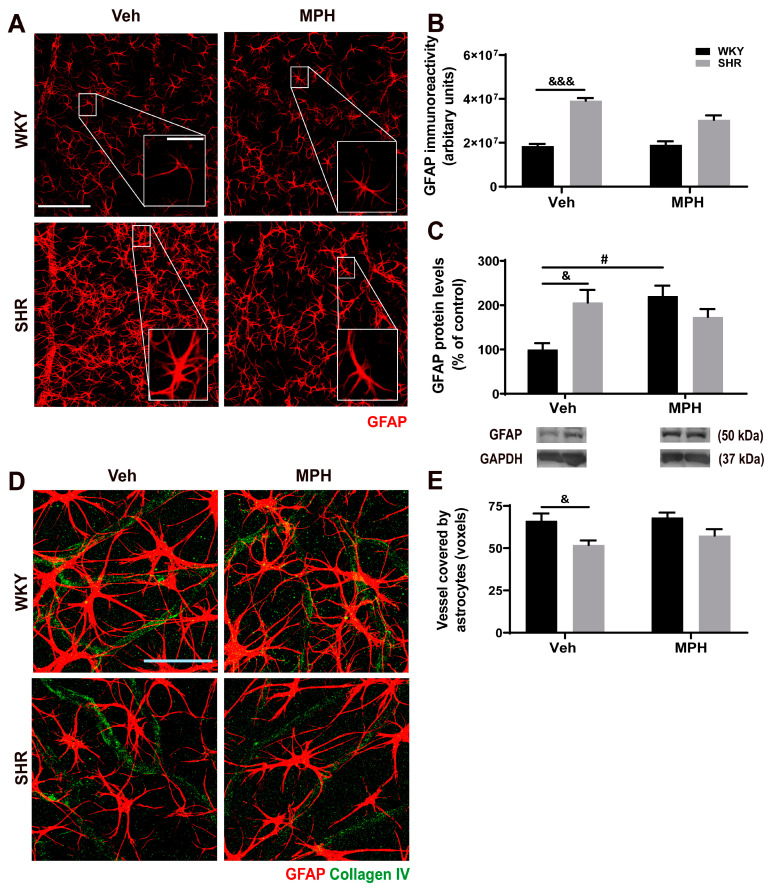
Astrogliosis in ADHD animal model and impact of MPH on astrocytic response. (**A**) Representative confocal images of wholemounts labeled for the astrocyte-specific glial fibrillary acidic protein (GFAP) and (**B**) quantification of GFAP immunostaining. Scale bars: 50 and 200 µm. (**C**) Protein levels and representative western blot images of GPAP (50 kDa) and GAPDH (37 kDa). (**D**) Representative confocal images of wholemounts co-labeled for Collagen IV (green) to identify blood vessels and the astrocyte-specific glial fibrillary acidic protein (GFAP). Scale bar: 50 µm. (**E**) Quantification of the area fraction per field of Collagen IV and GFAP positive staining, along with their respective co-localization with vessels. The ADHD model presented an increase in GFAP immunoreactivity and protein levels in the retina. The ADHD model showed a decrease in the number of GFAP/Collagen IV co-localized voxels, demonstrating that retinal vessel coverage by astrocytes decreased. MPH exposure had no effects on such alterations. Results are shown as mean ± SEM or mean ± SEM, as % of control, *n* = 3 animals for each condition. ^&^ *p* < 0.05, ^&&&^ *p* < 0.001, WKY Veh vs. SHR Veh; ^#^ *p* < 0.05, WKY Veh vs. WKY MPH, using two-way ANOVA followed by Tukey’s test or Kruskal-Wallis test followed by Dunn’s post-test.

In the retina, astrocytes have a close and important relationship with neurons and the blood vessels’ integrity [33]. Thus, we analyzed possible alterations in the retinal astrocytes in the ADHD animal model (Figure 7). We observed a significant increase in GFAP immunoreactivity (3.9 × 10^7^ ± 1.2 × 10^6^ arbitrary units; Figure 7B) in the retina wholemounts in this model compared to the controls (1.8 × 10^7^ ± 1.0 × 10^6^ arbitrary units). The total protein levels of GFAP in the retina also showed a significant astrogliosis in the SHR animals (205.9 ± 28.7% of the control) (Figure 7C) compared to the WKY rats. Treatment of the SHR animals with MPH did not significantly alter the GFAP immunoreactivity and protein levels compared to the non-treated SHR animals (Figure 7A–C). The MPH treatment upregulated the GFAP protein levels in the control rats (220.3 ± 23.6% of the control), but not the GFAP immunoreactivity in the retina wholemounts, compared to the non-treated animals.

The vessel coverage by astrocytes, which has an important role in vessel integrity and is affected by the astrocyte reactivity [29], was evaluated in the retina wholemounts (Figure 7D). The number of GFAP/Collagen IV co-localized voxels was significantly decreased in the retinas of the ADHD model (Figure 7E) compared to the controls, which indicates a significant decrease in the vessel coverage by astrocytes (66.1 ± 4.347% and 51.9 ± 2.69% of co-localized voxels for WKY Veh and SHR Veh, respectively). The treatment with MPH had no impact both in ADHD model and in the control rats. These results show that the ADHD model presents retinal astrogliosis, which impacted the vessel coverage by astrocytes. In the ADHD condition, the MPH treatment had no significant effects in the GFAP immunoreactivity and levels, as well as in the vessel coverage, in the retina. However, MPH per se increased the astrocyte reactivity in the retinas of the control rats.

**Figure 8:**
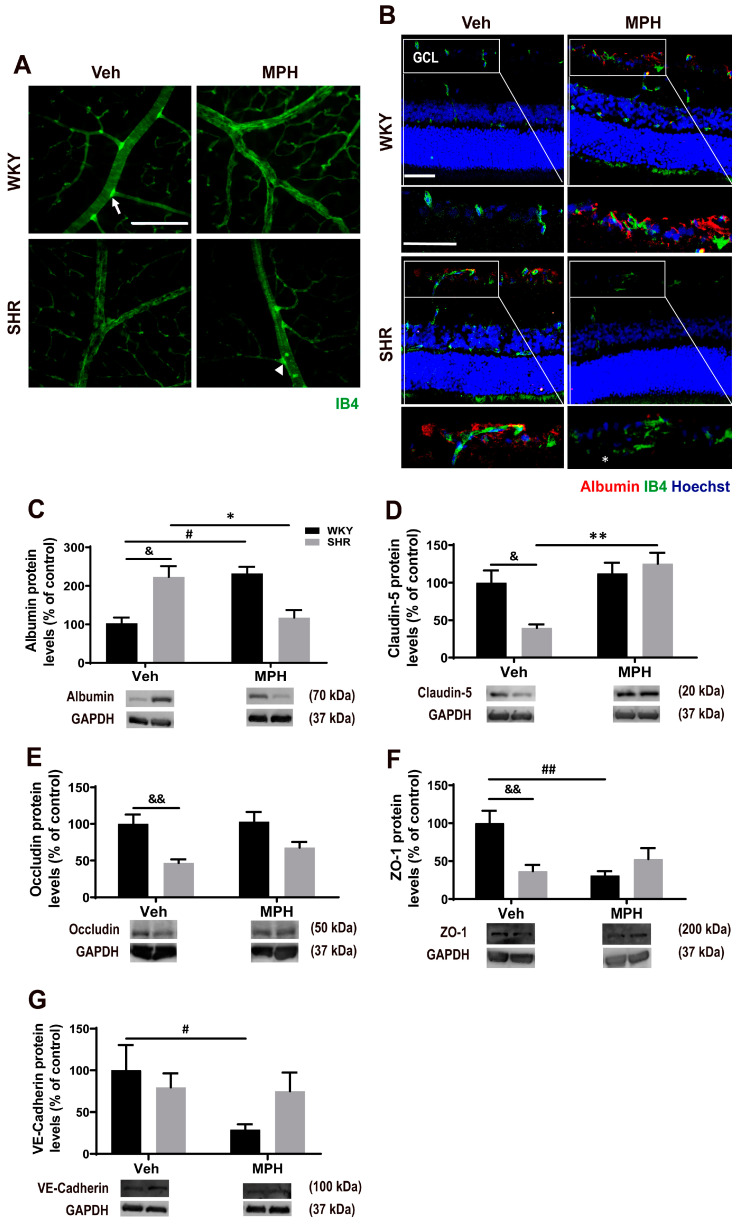
MPH effects on retinal vasculature in ADHD model and control animals. (**A**) Representative confocal images of wholemounts labeled for endothelial cells (Isolectin GS-IB4, green). Scale bar: 100 µm. (**B**) Representative confocal images of retinal cryosections co-labeled for endothelial cells (Isolectin GS-IB4, green) and albumin (red). Scale bars: 50 µm. (**C**) Quantification and representative western blot images of albumin (70 kDa) and GAPDH (37 kDa). Control retinas presented a clear delineation of the vessels and defined branching points (arrows). ADHD model presented a nonuniform staining of IB4 and undefined branching points. MPH treatment per se induced a similar profile in control rats but seems to partially rescue vessel integrity in ADHD retinas (arrowhead). A significant increase in albumin staining and protein levels were observed in ADHD model and MPH had a beneficial effect. (**D**–**G**) Quantification and representative western blot images of (**D**) claudin-5 (20 kDa), **(E)** occludin (50 kDa), (**F**) zonula occludens protein 1 (ZO-1, 200 kDa) and (**G**) VE-Cadherin (100 kDa) and GAPDH (37 kDa). ADHD model presented a downregulation of tight junction proteins. In control rats, MPH downregulated VE-cadherin and ZO-1 levels. Results are shown as mean ± SEM, as % of control, *n* = 2–3 animals for each condition. ^&^ *p* < 0.05, ^&&^ *p* < 0.01, WKY Veh vs. SHR Veh; ^#^ *p* < 0.05, ^##^ *p* < 0.01, * *p* < 0.05, ** *p* < 0.01 compared to the respective vehicle strain (Veh), using two-way ANOVA followed by Tukey’s test.

The integrity of the blood-retinal barrier (BRB) is essential for normal visual function [33]. The reactivity of astrocytes wrapping the retinal capillaries can have a deleterious impact on maintaining the inner BRB properties and function. Thus, considering the astrogliosis observed in the retinas of the SHR rats, we analyzed possible alterations in the retinal vasculature. The retina wholemounts were stained for isolectin GS-IB4 (Figure 8A), a marker of endothelial cells [34], to analyse potential retinal vessel alterations with more detail. In the control retina, it was possible to observe a clear delineation of vessels and defined branching points (arrow; Figure 8A). On the contrary, the ADHD model presented a non-uniform staining pattern of IB4 with no clear branching points suggesting a disorganization of the endothelial cells and potential vessel disruption. In the ADHD retinas, the MPH treatment seemed to partially rescue vessel integrity (arrowhead; Figure 8A). As already seen in the other parameters before, MPH per se affected vessel integrity in the retinas of the control rats. Thus, to further analyze the vessel integrity, the retinal cryosections were stained for albumin (Figure 8B), a high molecular weight protein that under normal physiological conditions does not cross the BRB. The protein levels of albumin in the retinal parenchyma were also quantified by western blot (Figure 8C). The ADHD animal model presented a significant increase in albumin staining (Figure 8B), particularly at the GCL (where inner capillaries are typically located), and albumin protein levels (244.3 ± 32.3% of the control; Figure 8C), compared to the control animals. In the SHR rats, MPH had a beneficial effect by significantly decreasing the albumin levels (117.6 ± 19.78% of control) compared to its respective strain (SHR Veh). Regarding the MPH effect per se, it promoted the disruption of BRB in the control animals with a significant upregulation of albumin immunostaining (Figure 8B) and protein levels (251.0 ± 18.2% of the control; Figure 8C).

Additionally, to explain the BRB hyperpermeability observed, we hypothesized that the retinal endothelial junction proteins, tight (TJs) and adherens junctions (AJs), that are responsible for controlling the paracellular transport pathway across BRB might be altered. Thus, we assessed the protein levels of TJs (Figure 8D–F) and AJs (Figure 8G) by western blot. Concerning TJs, we detected a significant downregulation of claudin-5, occludin and ZO-1 proteins (39.8 ± 4.6%, 46.7 ± 5.0% and 36.8 ± 8.1% of the control, respectively) in the ADHD model compared to the controls, but no alterations were identified on VE-cadherin levels (Figure 8G). The treatment of the SHR animals with MPH completely prevented the decrease in the claudin-5 levels (125.4 ± 14.4% of the control; Figure 8F), but MPH was unable to recover the levels of occludin and ZO-1. In the WKY rats, MPH decreased the protein levels of ZO-1 and VE-cadherin (31.1 ± 5.7% and 29.1 ± 6.4% of the control, respectively), but did not affect the claudin-5 and occludin protein levels. In sum, these results demonstrate that the BRB barrier is compromised in the ADHD model, thus increasing the retinal vessel permeability. In particular, the tight junction proteins claudin-5, occludin, and ZO-1 are downregulated in this model. The MPH treatment was able to prevent the BRB permeability and claudin-5 decrease in the SHR animals. However, in the control rats, MPH per se negatively affected the inner BRB integrity, increasing the barrier permeability, also decreasing the ZO-1 and VE-cadherin levels.

## 4. Discussion

The present study provides new evidence on both ADHD pathophysiology and the impact of MPH, the first-line pharmacological treatment for this condition. Specifically, we demonstrated that the ADHD animal model presents retinal alterations and that MPH was able to rescue some of these features. Conversely, MPH had a negative impact in non-pathological conditions. In detail, our results show that the ADHD animal model presented retinal thinning. These results are in agreement with a previous study by Li et al., 2020 [35], where they showed that in animals with 10 weeks of age, the outer nuclear layer thickness of SHR was significantly thinner than that of WKY. Hergüner and collaborators [36] also showed that ADHD children presented thinner nasal RNFL, being this positively correlated to the ADHD symptoms severity. The evaluation of RNFL and GCL thickness as possible biomarkers in children and adolescents diagnosed with ADHD has been described [37,38] once the RNFL contains RGCs with unmyelinated axons, which are considered to be an extension of the cerebral gray matter. The development of ADHD involves a delay in brain maturation, including a reduced volume of cerebral gray matter, which is linked to a disruption in the neurological pathways [39]. ADHD children presented a significant reduction in RNFL thickness, although the reduced GCL thickness in these children was not statistically different from the controls [36]. In our preclinical study, using an ADHD animal model, we successfully identified these same alterations in the RNFL and GCL. Curiously, as we show in our study, Isik and Kaygisiz [40] did not find the beneficial or deleterious effects of the MPH treatment on the retinal thickness in ADHD conditions. Moreover, we evaluated for the first time the possible alterations in several retinal layers, demonstrating that the retinal layers assessed, except the IS/OS, are thinner in the ADHD animal model. The decrease in the number of the photoreceptors and RGCs observed in the ADHD retinas suggests that retinal thinning is associated with cell loss and cannot be prevented by MPH. To the best of our knowledge, this is the first study that explored the effects of MPH on retinal structure in ADHD conditions.

One of the hypotheses for ADHD etiology relies on the impairment in the dopaminergic system [41,42], and it is well established that dopamine plays a critical role in retinal function [43]. Adults with ADHD present an increase in the background noise in pattern ERG (reflecting non-stimulus-driven RGCs activity) [16]. Yet, this alteration was described only in adults, and there are no studies regarding children and adolescents. Another important clinical feature described in ADHD children and adolescents is the alteration in color perception [44]. Interestingly, in pathological conditions characterized by an impairment in the dopaminergic system, such as Parkinson’s disease, visual acuity and color vision deficits have also been reported in both animal models [45,46] and PD patients [47]. Similarly, we observed that the ADHD model presents visual function impairment, with a deficit in the photoreceptors/bipolar cell circuits, and RGCs circuits. Thus, the impairment in these circuits demonstrates that the ADHD model has retinal function impairment that may be related to ADHD features, namely: distractibility and inattention [46] and reduced visual acuity [48], which are also described in ADHD children and adolescents [49,50]. However, we did not detect any alterations in the photopic b-wave in the ADHD conditions, which suggests that the bipolar cell’s function impairment is less subtle when the retina is light-adapted.

Recent studies also demonstrated that the downregulation of the glutamate receptors (GluR) might contribute to ADHD pathogenesis [51]. It is described that the behavioral abnormality caused by GluR deficiency may depend on dopamine receptors. In turn, the glutamate system may regulate dopamine-related behavioral problems [52]. Importantly, Cheng and collaborators [53] demonstrated that the SHR rats present a decrease in AMPAR-mediated synaptic transmission in the pre-frontal cortex (PFC), consistent with the hypothesis that ADHD is also a hypoglutamatergic condition. Accordingly, we observed alterations in the glutamatergic system by showing a downregulation in VGlut1 immunoreactivity in ADHD retinas. The decrease in VGlut1 could interfere with the synaptic signaling of the photoreceptors and bipolar cells due to changes in excitatory synapses along the rod and cone pathways [54], which can correlate with the ERG deficits that we also found in our ADHD animal model. Regarding the MPH effect in the ADHD model, it did not prevent retinal thinning, photoreceptor and RGCs loss, visual function impairment nor decreased VGlut1 levels. Taking into consideration the most well-known mechanism of action of MPH as a blocker of dopamine and noradrenaline transporters with a consequent increase in these monoamines in the synaptic cleft [5], our results may suggest that this specific effect of MPH is not sufficient to ameliorate vision function alterations in the ADHD model.

Another key objective of this study was the evaluation of the effect of MPH per se on the retina, in the control conditions. It is also important to evaluate the consequences of MPH misuse. Although MPH did not significantly alter the retinal structure, at the cellular level, there were differences. The number of cone and rod photoreceptors and RGCs decreased considerably. Moreover, MPH impaired the scotopic photoreceptor/bipolar cells pathway, and the synaptic protein synapsin and PSD95 levels. On the other hand, no alterations were found in the bipolar cell circuits under photopic conditions and on the RGCs’ function, suggesting that misuse of MPH may impact differently the dark versus light synaptic processing between the outer and the inner retina. Interestingly, an acute intraperitoneal administration of MPH (2 mg/kg) was shown to improve sensory processing and visual performance in rats [19,55], demonstrating that acute exposure to a clinically relevant dose of MPH could indeed act as a cognitive enhancer. In contrast, chronic exposure may lead to deleterious effects on the retina. Yang and collaborators [56] showed that chronic low doses of methamphetamine (another stimulant, 0.5–1 mg/kg) negatively impact photoreceptors and bipolar cells’ response in dark-adapted conditions.

Besides neurons, the retina is constituted by other cell types that also play an important role in its (dys)function. Microglial-mediated neuroinflammation is known to be involved in several retinal degenerative diseases [57], and microglial reactivity was previously associated with the degeneration of the photoreceptors [58] and RGCs [59,60]. Concerning ADHD, using a PET tracer for measuring activated microglia in the dorsolateral prefrontal cortex (DLPFC), Yokokura and collaborators [61] showed that microglia reactivity is correlated with worse performance score on tasks of processing speed and attention in individuals with ADHD. Here we demonstrated that the ADHD model presents an increase in microglial reactivity in the retina concomitant with the downregulation of CX3CR1. The fractalkine receptor CX3CR3 has been described to play an essential role in microglia-neuron communication, and CX3CR1-deficiency has been correlated with an increase in microglia reactivity, which culminates in retinal neurotoxicity and neuronal death [56,60]. Thus, microglia reactivity could play a key role in the photoreceptors and the RGCs’ degeneration observed in this study. The beneficial effects of the MPH treatment on microglial activation and CX3CR1 levels observed in the ADHD model are in accordance with our previous study, in the brain [4], where we demonstrated a decrease in microglial reactivity in the ADHD model after treatment with MPH (1.5 mg/kg). However, MPH per se also led to an increase in microglial reactivity and a downregulation of CX3CR1, which once again corroborates our previous findings in the brain [4] and reinforces the concerns regarding the impact of chronic exposure to MPH under non-ADHD conditions.

Astrocyte distribution in the retina is mainly associated with blood vessels. Astrocytes send processes to wrap both blood vessels and axons [62]. In the ADHD model, it reports an increase in astrogliosis in the brain, specifically in the PFC [4], which is in accordance with our results. Likewise, corroborating findings described in this study, the increase in the astrocyte reactivity has been pointed out as an important key player in retinal degeneration [62], mainly due to the upregulation of the expression of various genes encoding cytokines, chemokines, and elements of the complement cascade. Furthermore, we observed a significant decrease in vessel coverage by astrocytes in the ADHD model, indicating an impairment of astrocyte function in the maintenance of the integrity of the inner BRB [62]. Our results demonstrate that the ADHD model also presents an increase in vascular permeability since it identified an increase in albumin leakage, vessel disruption, and a decrease in TJ protein levels. Interestingly, Li and collaborators [35] have previously shown that SHR present more tortuous retinal vessels compared to WKY, which suggest vascular alterations. Regarding the effect of MPH per se on the retina in several parameters assessed in this model, the treatment with MPH had no effects on the astrogliosis in the ADHD model. Nevertheless, it prevented the increase in vascular permeability in the SHR animals since the albumin levels were decreased, and the endothelial cell architecture partially recovered when compared to the ADHD animal model without treatment. The MPH beneficial effects may be related to the increase in the claudin-5 levels since this protein is highly expressed in the endothelial cells, is the most enriched TJ, and is essential for barrier establishment and maintenance [63].

The impact of stimulant misuse on astrocytes behavior and BRB integrity has been described. Indeed, astrocytes have been considered active players promoting drug-induced synaptic and circuit remodelling in the formation of cocaine memories [64], and METH administration led to neuronal loss, increased TNF and matrix metalloproteinase-14 levels, and decreased levels of retinal vascular endothelial surface molecules [65]. Regarding the impact of MPH on the control conditions, and in accordance with our previous study in the brain [4], the MPH treatment increased GFAP protein levels and albumin leakage in the control conditions, promoted endothelial cell disorganization, and downregulated VE-cadherin and ZO-1 levels. These observations are in accordance with our previous study, where we identified that MPH negatively impacts brain endothelial permeability by stimulating vesicular transport involving oxidative stress [6]. Studies with METH also described paracellular and transcellular transport impairment in endothelial cells [66,67]. Here we demonstrated that the misuse of MPH compromises BRB integrity. These results mimic the deleterious effects observed in PFC [4] and in the hippocampus of the control rats after administration of MPH (5 mg/kg) [7].

Since significant alterations were observed in microglia and astrocytes, and these glial cells are an important source of inflammatory mediators, we further characterized the inflammatory status of the retina in ADHD and the control conditions after the MPH treatment. Our results demonstrate that the ADHD model presents an increase in pro-inflammatory cytokines in the retina and an increase in the anti-inflammatory cytokine IL-4. It has been described that the increase in the IL-4 release by glial cells may be a mechanism to suppress the exaggerated pro-inflammatory response [68,69], namely the elevated levels of IL-6 and TNF [70], which seems to be in accordance with our results. Moreover, a pro-inflammatory environment plays an important role in retinal dysfunction [71], which is in accordance with the alterations in retinal function detected in the untreated ADHD animal model. Moreover, treatment with MPH decreased TNF and iNOS levels and showed a tendency for the same effect concerning IL-6. Moreover, the harmful effects of MPH on the levels of the inflammatory mediators in the control conditions are in accordance with the effects previously observed in the PFC [4]. The pro-inflammatory mediators derived from astrocytes are already described to induce microglial activation after METH exposure [72]. These observations lead us to suggest that in the retina, both microglia and astrocytes may be underlying the pro-inflammatory status triggered by MPH in the control rats. Moreover, since the retina is one of the tissues with the highest oxygen consumption, it is a primed environment for reactive oxygen species (ROS) generation [73]. Although in this study we did not specifically address the levels of ROS, we did observe an increase in iNOS and several pro-inflammatory cytokines, as previously detailed. It is well known that oxidative stress can favour the production of inflammatory cytokines, such as TNF and IL-6 [74]. In fact, we have previously shown that the ADHD rat model presented increased ROS levels, lipid peroxidation, and TNF in the brain (prefrontal cortex) [4]. MPH counteracted these alterations at the same concentration used in the present study (1.5 mg/kg/day). Thus, in a future study it would be interesting to explore in detail the oxidative status of the retina in ADHD conditions, as well as the impact of MPH on such alterations. Additionally, and since we only used males, we would like to investigate in the near future the possible sex differences regarding both brain and retinal features associated with ADHD and MPH use.

## 5. Conclusions

Our findings demonstrate that in the ADHD animal model, there are structural and functional alterations in the retina, which seem to be aligned with the visual alterations described in ADHD children and adolescents. The MPH treatment did not improve the retinal structural and functional impairment nor the decrease in neuronal cells observed, but partially rescued the inner BRB disruption, glial cells reactivity, and the pro-inflammatory profile in the ADHD model. Despite these significant protective effects of MPH in the ADHD model, under the control conditions, exposure to MPH had deleterious effects on the retinal physiology and on the integrity of the inner BRB, promoted glial cells reactivity, and induced a pro-inflammatory status. In sum, this study gives significant and new contribution to a better understanding of ADHD pathophysiology, specifically in an organ that has been overlooked in the field. Thus, our findings highlight the importance of the diagnosis and the consequences of the MPH treatment in ADHD, as well as the misuse of MPH in non-ADHD conditions regarding retina physiology.

## Data Availability

All data are included in the article and there is no additional data elsewhere.

## Supplementary Materials

The following supporting information can be downloaded at: https://www.mdpi.com/article/10.3390/antiox12040937/s1, Figure S1: In vivo electroretinography (ERG) responses on ADHD model and control conditions and the effect of MPH treatment, Table S1: List of primary antibodies used in western blot (WB) and immunohistochemistry (IHC) analysis, Table S2: List of secondary antibodies used in immunohistochemistry (IHC) analysis.
