# Supplementary material for: Attention-Deficit/Hyperactivity Disorder Animal Model Presents Retinal Alterations and Methylphenidate Has a Differential Effect in ADHD versus Control Conditions
Source: Antioxidants (Basel). 2023 Apr 15;12(4):937. doi: 10.3390/antiox12040937 (PMC10135983; doi:10.3390/antiox12040937)
Supplement: Supplementary file 1 [file antioxidants-12-00937-s001.zip › antioxidants-2286678-supplementary.pdf]

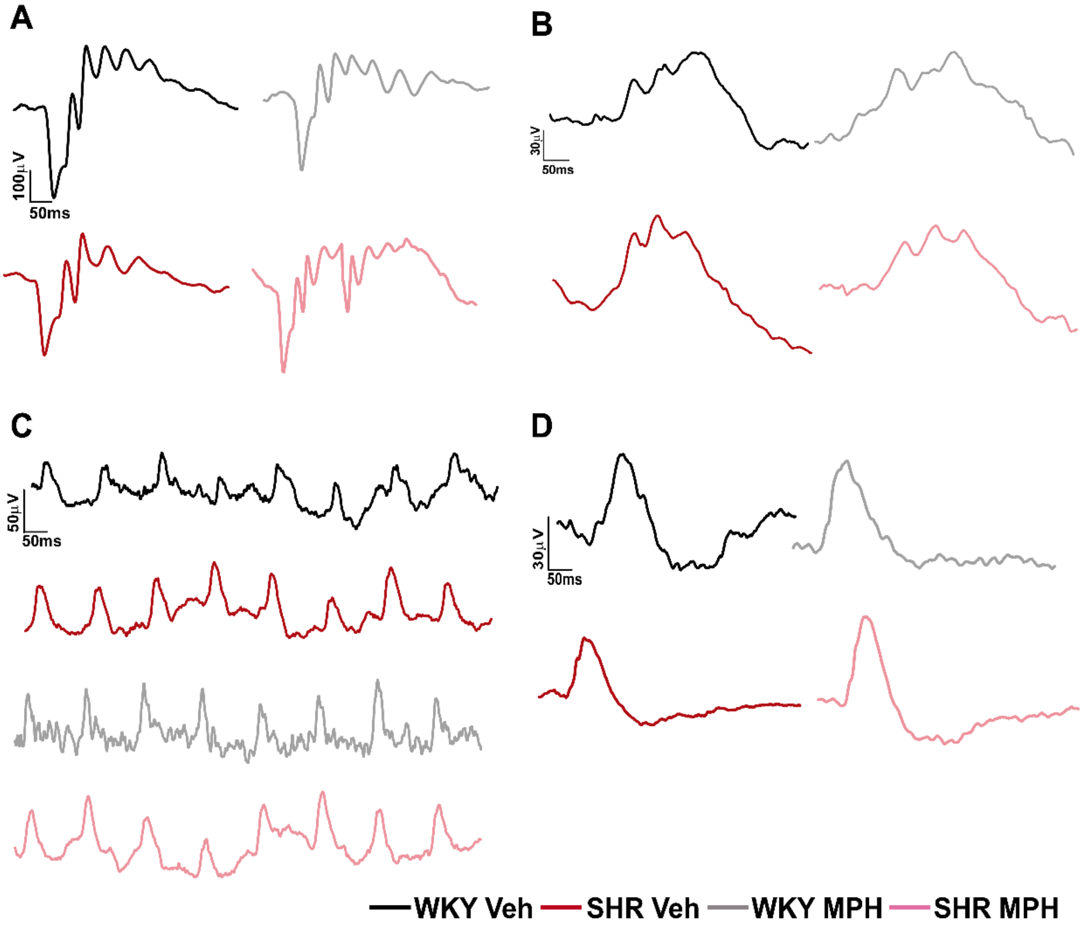

Figure S1. I*n vivo* electroretinography (ERG) responses on ADHD model and control conditions and the effect of MPH treatment. Representative traces of individual (A) scotopic, (B) photopic, (C) flicker, and (D) scotopic threshold response of WKY and SHR rats in basal conditions and after MPH treatment. Waveforms were elicited using flash light stimuli with different intensities (described in the material and methods section). ADHD model presented impaired photoreceptors and bipolar cells responses in the dark, and also cone photoreceptors and retinal ganglion cells dysfunction. MPH treatment had no impact on such alterations in the ADHD model, but MPH per se interfered with photoreceptors and bipolar cells response in control rats. MPH=Methylphenidate.

.**Table S1**: List of primary antibodies used in western blot (WB) and immunohistochemistry (IHC) analysis.

Antibody	Supplier	Cat. No	Dilution
Anti-Albumin	Bethyl Laboratories, Inc	A90-134A	1:2000
Anti-Arrestin	Millipore	AB15282	1:500
Anti-Claudin5	Thermo Fisher	34-1600	1:200
Anti-Collagen IV	Abcam, Cambridge, UK	Ab6586	1:200
Anti-CD68	Abcam	Ab53444	1:500
Anti-CX3CR1	Abcam	Ab8021	1:500
Anti-GFAP	Sigma-Aldrich, St. Louis, MO, USA	G9269	1:2000
Anti-GFAP-Cy3-conjugated	Sigma-Aldrich, St. Louis, MO, USA	G9205	1:1000
Anti-Iba1	Wako	019-19741	1:1000
Anti-IB4, Alexa Fluor 647 conjugate	Invitrogen	132450	1:100
Anti-IL1β	Santa Cruz Biotechnology	Sc-7884	1:100
Anti-IL4	R&D Systems	MAB504	1:200
Anti-IL6	R&D Systems	406-ML/CF	1:200
Anti-IL10	Invitrogen	PA5-95561	1:200
Anti-iNOS	Novus Biologicals	NB300-605	1:500
Anti-Occludin	Invitrogen	71-1500	1:200
Anti-PSD95	Cell Signalling	D27E11	1:5000
Anti-RBPMS	Abcam	Ab194213	1:500
Anti-Rhodopsin	Millipore	MABN15	1:500
Anti-Synapsin	Synaptic Systems	106 011	1:10000
Anti-TNF	Abcam	Ab6671	1:100
Anti-VE Cadherin	Santa Cruz Biotechnology	Sc-6458	1:200
Anti-VGlut1	Abcam	Ab227805	1:500
Anti-ZO1	Invitrogen	61-7300	1:200

Abbreviations: CD68, cluster of differentiation 68; CX3CR1, fractalkine receptor; GFAP, glial fibrillary acidic protein; Iba-1, calcium-binding protein; IB4, Isolectin GS-IB4; IL-1β, interleukine-1beta; IL-4, interleukine-4; IL-6, interleukine-6; IL-10, interleukine-10; iNOS, inducible nitric oxide synthase; PSD95, Postsynaptic density protein-95; RBPMS, RNA-binding protein with multiple splicing; TNF, tumor necrosis factor; VGlut1, vesicular glutamate transporter 1; VE-cadherin, vascular endothelial cadherin; ZO-1, zonula occludens-1.

Table S2. List of secondary antibodies used in immunohistochemistry (IHC) analysis.

Antibody	Supplier	Cat. No	Dilution
Alexa Fluor anti-goat 594	Invitrogen	A11058	1:500
Alexa Fluor anti-mouse 568	Molecular Probes	A10037	1:500
Alexa Fluor anti-rabbit 568	Invitrogen	A31458	1:500
Alexa Fluor anti-rat 488	Invitrogen	A-21208	1:500

Antibody	Supplier	Cat. No	Dilution
Alexa Fluor anti-goat 594	Invitrogen	A11058	1:500
Alexa Fluor anti-mouse 568	Molecular Probes	A10037	1:500
Alexa Fluor anti-rabbit 568	Invitrogen	A31458	1:500
Alexa Fluor anti-rat 488	Invitrogen	A-21208	1:500
